# The neutrophil/lymphocyte ratio as a predictor of peritoneal metastasis during staging laparoscopy for advanced gastric cancer: a retrospective cohort analysis

**DOI:** 10.1186/s12957-019-1651-3

**Published:** 2019-06-25

**Authors:** Naohiko Nakamura, Shinichi Kinami, Yoritaka Fujii, Seiko Miura, Jun Fujita, Daisuke Kaida, Yasuto Tomita, Takashi Miyata, Hideto Fujita, Nobuhiko Ueda, Yasuo Iida, Takeo Kosaka

**Affiliations:** 1 Department of Surgical Oncology, Kanazawa Medical University Hospital, 1-1 Daigaku, Uchinada, Kahoku, Ishikawa 920-0293 Japan; 2 Department of Mathematics, Division of General Education, Kanazawa Medical University Hospital, 1-1 Daigaku, Uchinada, Kahoku, Ishikawa 920-0293 Japan

**Keywords:** Gastric cancer, Staging laparoscopy, Neutrophil/lymphocyte ratio

## Abstract

**Background:**

The use of staging laparoscopy (SL) has become widespread in patients with advanced gastric cancer (GC). This study aimed to evaluate the predictive value of the neutrophil/lymphocyte ratio (NLR) for the presence of peritoneal metastasis during staging laparoscopy in patients with advanced GC.

**Methods:**

This retrospective analysis was performed in 35 patients with advanced GC who underwent SL at Kanazawa Medical University Hospital between January 2009 and December 2017. Clinicopathological characteristics were examined and multivariate analyses were performed to identify preoperative laboratory parameters that were independently associated with the presence of peritoneal metastasis or cytological malignancy (P/CY positive) during SL.

**Results:**

A P/CY-positive result was confirmed during SL in 16 patients (45.7%). Patients with type 4 or diffuse type 3 tumors showed a significantly higher P/CY-positive rate than those with other tumor types (58.3% vs. 18.2%, *P* = 0.02). In the univariate analysis for preoperative laboratory parameters, NLR (*P* < 0.0001) and total protein (*P* = 0.03) and albumin (*P* = 0.04) levels were significantly correlated with a P/CY-positive result. On multivariate analysis, NLR was significantly correlated with a P/CY-positive result (*P* = 0.0002). In patients with type 4 or diffuse type 3 tumors, a high NLR (> 3.5) was associated with a significantly higher P/CY-positive rate than a low NLR (≤ 3.5) (83.3% vs. 33.3%, *P* = 0.01). Moreover, in patients without type 4 or diffuse type 3 tumors, the P/CY-positive rates were 100% and 0% in patients with NLR > 3.5 and NLR ≤ 3.5, respectively.

**Conclusions:**

The preoperative NLR was a significant independent predictor of the presence of peritoneal metastasis during SL. Regardless of tumor type, patients with a high NLR could be reasonable candidates for SL. On the other hand, non-diffuse type tumor accompanied by a low NLR may not need to undergo SL.

## Background

Gastric cancer (GC) is the fifth most common malignancy and the second most common cause of cancer mortality worldwide [1]. The curative treatment for advanced GC consists of gastrectomy with perioperative chemotherapy in Western countries [2, 3]. Even in stage IV GC, curative resection by conversion gastrectomy following systemic chemotherapy could improve the prognosis [4]. In such an advanced stage of GC, accurate evaluation of peritoneal metastasis is essential for providing optimal treatment because the GCs in most patients who have exhibited peritoneal metastasis are not suitable for curative resection by gastrectomy. Several imaging methods are employed in the staging of GC. A recent meta-analysis showed that computed tomography has good accuracy in staging GC, but the detection of peritoneal metastasis remains to have low sensitivity and specificity [5]. In these situations, staging laparoscopy (SL) has played a cardinal role in the investigation of treatment strategies for patients with advanced GC in recent years [6].

SL for GC has been particularly used in patients with locally advanced GC, candidates for neoadjuvant therapy, and those suspected with peritoneal metastasis [7]. Several studies have demonstrated the superiority of SL over conventional imaging tests for detecting peritoneal metastasis [8, 9]. Although indicating SL based on preoperative clinicopathological factors is still controversial, patients with type 4 or diffuse-type tumors could have a high potential for peritoneal disease spread and be considered as more-suitable candidates for SL [10, 11]. However, SL is an invasive procedure and is accompanied by a risk of adverse events for the patients. Additionally, SL should not be recommended on a routine basis because of its cost. Therefore, noninvasive clinical parameters that can predict the presence of peritoneal metastasis are urgently needed to avoid unnecessary laparoscopy.

In recent years, there is a rapidly growing interest in the association between the neutrophil/lymphocyte ratio (NLR) and the clinical outcomes of upper gastrointestinal cancers. A retrospective analysis of GC patients who underwent gastrectomy has observed an association between a high NLR and poor survival, tumor depth, and peritoneal metastasis [12]. The NLR is calculated as the neutrophil count divided by the lymphocyte count and is easily measurable in a routine preoperative examination for GC patients. Thus, we conceived that the preoperative NLR could help in narrowing down the selection of candidates for SL. In this context, this study aimed to evaluate the predictive value of the NLR for the presence of peritoneal metastasis or cytological malignancy (P/CY positive) during SL in patients with advanced GC.

## Materials and methods

### Patients

This retrospective analysis was performed in 35 patients with advanced GC who underwent SL at Kanazawa Medical University Hospital between January 2009 and December 2017. For the clinical staging for GC, all the patients underwent abdominal computed tomography scan and positron emission tomography. The candidates for SL in this study were patients with advanced gastric cancer who were suspected to have peritoneal or severe lymph node metastasis during the preoperative imaging examination or who were shown to exhibit type 4 or diffuse-type tumors on upper gastrointestinal endoscopy. In addition to these findings, during the preoperative examinations, we performed SL in patients who could potentially undergo curative resection or neoadjuvant chemotherapy, if peritoneal metastasis was not detected through the SL. We obtained informed consent from the patients. This study was approved by the Medicine Ethics Committee of Kanazawa Medical University.

### Clinicopathological evaluations

Data on clinical characteristics, gender, age, and body mass index were extracted from our hospital’s database. The tumors of all patients were histologically confirmed as adenocarcinomas and clinically staged according to the 7th edition of the American Joint Committee on Cancer (AJCC) [13] based on tumor location, macroscopic tumor type, depth of tumor invasion (T), extent of lymph node metastasis (N), and distant metastasis (M). We defined type 3 GC that was located from the upper to lower stomach as diffuse type 3 tumor. We collected the results of the blood examination before SL, including white blood cell (WBC) count, fraction of neutrophils and lymphocytes in WBC differentiation, NLR, hemoglobin level, serum platelet count, and levels of C-reactive protein (CRP), total protein (TP), albumin, cholinesterase, carcinoembryonic antigen (CEA), carbohydrate antigen 19-9 (CA19-9), and carbohydrate antigen 125 (CA125).

### Surgical procedures and pathological evaluations

SL was performed as follows by gastrointestinal surgeons: (1) a small subumbilical incision was made, and a 12-mm trocar was inserted; (2) 50–100 ml of saline was used to irrigate the pouch of Douglas and then was aspirated for cytological examination; (3) the visible surface of the stomach, liver, diaphragm, omentum, and peritoneum was systematically explored in search of malignant deposits. In case of suspicious macroscopic lesions, biopsies were performed and sent for histological review. A P/CY-positive result was defined when peritoneal dissemination or presence of adenocarcinoma cells in the cytology specimens was histologically confirmed by pathologists.

### Statistical analysis

Data were expressed as *n* (%) or medians (range). Continuous variables were compared using Student’s *t* test, while categorical variables were compared using the *χ*^2^ test. All *P* values were two-sided, and differences with a *P* value of < 0.05 were considered statistically significant. A logistic regression model was used to identify clinical factors that were independently associated with a P/CY-positive result. Variables that were associated with a P/CY-positive result at *P* ≤ 0.10 in the univariate analysis were included in the multivariate analysis. The diagnostic performance of the NLR with regard to the P/CY-positive rate was evaluated using multivariate receiver operating characteristic (ROC) curve analysis. All statistical analyses were performed using JMP software version 8.0 (SAS Institute, Cary, NC, USA).

## Results

### Patient characteristics

The median age of the study population was 66.5 years, and approximately two thirds of patients were male (Table 1). Before SL, the median value of the NLR was 2.3. Preoperative examinations indicated that 88.6% of patients had type 3 or 4 GC, 80.0% had cT4 disease, and 51.4% had diffuse-type tumors. The proportion of patients who were clinically diagnosed with stage IV disease was 34.4%. Among 28 patients who were clinically diagnosed as not having peritoneal metastasis before SL, 10 (35.7%) were surgically evaluated as P/CY positive during SL (Table 2). Among patients who were clinically suspected with peritoneal metastasis, 85.7% were confirmed P/CY positive by SL. Only one patient was P/CY negative during SL despite being suspected with peritoneal metastasis during the preoperative examination.

**Table 1:** Patient characteristics

	*n* = 35
Gender (male)	23 (65.7%)
Age	69 (30–86)
BMI	20.9 (15.5–30.9)
White blood cell (μl)	6340 (3140–12,960)
Neutrophil (μl)	4150 (1380–10,640)
Lymphocyte (μl)	1310 (690–3220)
NLR	2.3 (0.8–7.6)
Hemoglobin (g/dl)	12.2 (7.3–15.7)
Platelet count (× 10^4^/μl)	29.6 (13.1–61.4)
CRP (mg/dl)	0.1 (0.1–13.5)
Total protein (g/dl)	6.7 (4.5–8.2)
Albumin (g/dl)	3.8 (2.1–4.4)
Cholinesterase (U/l)	240 (107–410)
CEA (ng/ml)	2.4 (0.5–1380)
CA19-9 (U/ml)	19.8 (0.5–159.5)
CA125 (U/ml)	11.5 (4.0–247.3)
Tumor differentiation (poorly or signet)	10 (28.6)
Tumor type (3, 4)	31 (88.6%)
Tumor location (UML)	18 (51.4%)
T 4^a^	28 (80.0%)
N > 2^a^	18 (51.4%)
clinical Stage II/III/IV^a^	7 (20.0%)/16 (45.7%)/12 (34.3%)

Values are in *n* (%) or medians (range)

^a^7th edition of the American Joint Committee on Cancer (AJCC) [13]

*BMI*, Body mass index; *NLR*, neutrophil/lymphocyte ratio; *CRP*, C-reactive protein; *CEA*, carcinoembryonic antigen; *CA19-9*, carbohydrate antigen 19-9; *CA125*, carbohydrate antigen 125

**Table 2:** Clinical and surgical diagnoses for peritoneal metastasis of cancer

	Preoperative peritoneal metastasis (+) (*n* = 28)	Preoperative peritoneal metastasis (−) (*n* = 7)
P and CY negative at SL	18 (64.3%)	1 (14.3%)
P or CY positive at SL	10 (35.7%)	6 (85.7%)

Values are in *n* (%)

*SL*, staging laparoscopy; *P*, peritoneal metastasis; *CY*, cytology

### Proportion of P/CY-positive patients according to clinical features of GC

The correlation between the P/CY-positive rates at SL and the clinical stage or microscopic tumor type in the preoperative examinations was analyzed (Table 3). In patients with type 4 or diffuse type 3 GC, 58.3% were P/CY positive during SL, and the proportion was significantly higher than that in patients with other tumor types (58.3% vs. 18.2%, *P* = 0.02). Meanwhile, there was no significant difference in P/CY-positive rates between clinical T and N factors.

**Table 3:** Correlation between the preoperative-clinical features of GC and the P/CY-positive rates

	P/CY positive	*P* value
Clinical T^a^		
T3 ≧ (*n* = 7)	2 (28.6%)	0.31
T4 (*n* = 28)	14 (50.0%)	
Clinical N^a^		
N1 ≧ (*n* = 17)	10 (58.8%)	0.13
N2 ≦ (*n* = 18)	6 (33.3%)	
Macroscopic tumor type		
Others (*n* = 11)	2 (18.2%)	0.02
Type 4 or diffuse type 3 (*n* = 24)	14 (58.3%)	
Presence of paraaortic lymph node metastasis		
(−) (*n* = 29)	14 (48.3%)	0.50
(+) (*n* = 6)	2 (33.3%)	

Values are in *n* (%)

^a^7th edition of the American Joint Committee on Cancer (AJCC) [13]

*GC*, gastric cancer; *P/CY*, peritoneal metastasis or cytological malignancy

### Correlation between preoperative parameters and P/CY-positive results

**Fig. 1:**
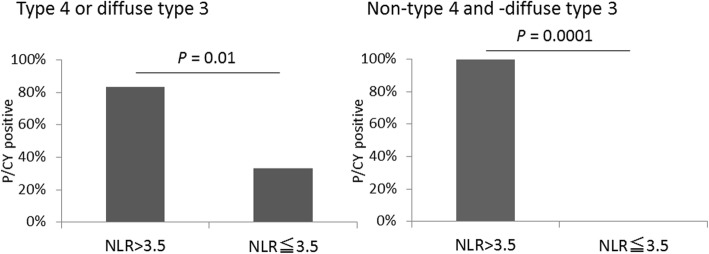
Correlation between NLR and peritoneal metastasis according to macroscopic tumor type. In patients with type 4 or diffuse type 3 tumors, 83.3% of those with NLR > 3.5 and 33.3% of those with NLR ≤ 3.5 had peritoneal metastases or malignant cells in the cytology specimens (P/CY positive). In patients without type 4 or diffuse type 3 tumors, 100% of those with NLR > 3.5 and 0% of those with NLR ≤ 3.5 were P/CY positive

**Fig. 2:**
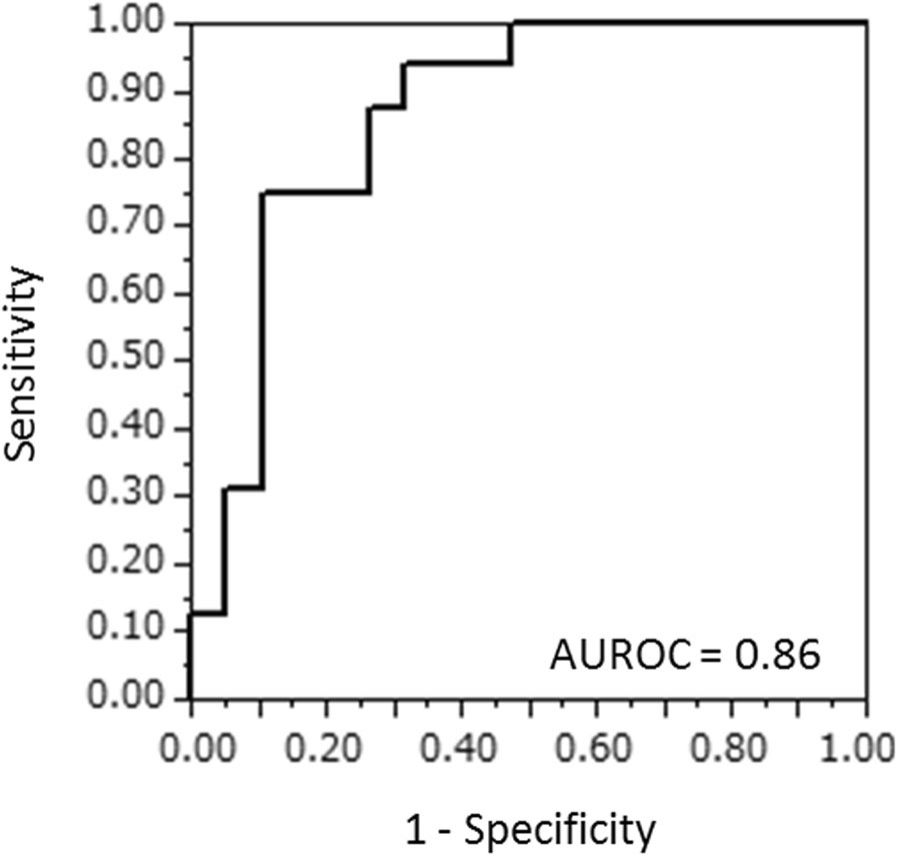
Diagnostic performance of NLR was evaluated by multivariate receiver operating characteristic (ROC). The area under the ROC curve (AUROC) of the NLR between the P/CY-positive and P/CY-negative results was 0.86. The best cutoff point of NLR was 3.7

Univariate analysis showed that the NLR (*P* < 0.0001), TP (*P* = 0.03), albumin (*P* = 0.04) levels, and type 4 or diffuse type 3 GC (*P* = 0.02) were significantly correlated with a P/CY-positive result during SL (Table 4). The P/CY-positive rates were 85.7%, 66.7%, 75.0%, and 58.3% in patients with NLR > 3.5, TP < 6.5 g/dl, albumin < 3.5 g/dl, and type 4 or diffuse type 3 GC, respectively. Multivariate analysis showed that NLR was significantly correlated with a P/CY-positive result (*P* = 0.0006). On the other hand, there was no significant difference in the predictive values of the tumor markers CEA, CA19-9, and CA125. Among patients who were not diagnosed with peritoneal metastasis in the preoperative examinations, the P/CY-positive rates were 80% and 11.1% in patients with NLR > 3.5 and NLR ≤ 3.5, respectively (*P* = 0.003). In patients with type 4 or diffuse type 3 tumors, the P/CY-positive rates were 83.3% and 33.3% in those with NLR > 3.5 and NLR ≤ 3.5, respectively (Fig. 1). Moreover, in patients without type 4 or diffuse type 3 tumors, the P/CY-positive rates were 100% and 0% in those with NLR > 3.5 and NLR ≤ 3.5, respectively. The area under the ROC curve of the NLR between the P/CY-positive and P/CY-negative results was 0.86 (Fig. 2). The best cutoff point of the NLR for distinguishing a P/CY-positive result was 3.7. At this cutoff point, the sensitivity and specificity were 0.75 and 0.89, respectively.

**Table 4:** Univariate and multivariate analyses to identify preoperative predictors of peritoneal metastasis

	Univariate analyses		Multivariate analyses	
	Odds ratio	*P* value	Odds ratio	*P* value
Age (> 70 years old)	2.2	0.27		
Gender (male)	0.5	0.28		
BMI (< 20)	2.2	0.28		
White blood cell (> 7000/μl)	0.6	0.51		
NLR (> 3.5)	25.5	< 0.0001	25.9	0.0006
Platelet count (< 30 × 10^4^/μl)	1.2	0.83		
Hemoglobin (< 12 g/dl)	1.7	0.43		
CRP (> 1 mg/dl)	4.2	0.21		
Total protein (< 6.5 g/dl)	4.7	0.03	7.8	0.08
Albumin (< 3.5 g/dl)	5.1	0.04	2.8	0.53
Cholinesterase (< 240 U/l)	1.1	0.88		
CEA (> 5 ng/ml)	1.1	0.93		
CA19-9 (> 37 U/ml)	2.2	0.28		
CA125 (> 35 U/ml)	3.9	0.12		
Type 4 or diffuse type 3	6.3	0.02	5.6	0.13
T4^a^	2.5	0.30		
N2 ≦^a^	0.35	0.13		
Stage IV^a^	2.2	0.29		

*BMI*, Body mass index; *NLR*, neutrophil/lymphocyte ratio; *CRP*, C-reactive protein; *CEA*, carcinoembryonic antigen; *CA19-9*, carbohydrate antigen 19-9; *CA125*, carbohydrate antigen 125

^a^7th edition of the American Joint Committee on Cancer (AJCC) [13]

## Discussion

Since the 1990s, SL has been performed for patients with resectable advanced GC, with a reported detection rate of metastatic disease of 21–31% [14, 15]. The use of SL has become widespread, but recently, its optimal indication restricts it to patients with a high risk of metastatic disease [16–18]. Such selective strategy can help avoid unnecessary laparoscopy for patients with resectable tumors and prevent potential complications of SL. Previously, patients with large infiltrating type 3 and type 4 tumors were reported as reasonable candidates for SL [10, 11, 18]. In our results, patients with type 4 or diffuse type 3 tumors showed higher P/CY-positive rates during SL. On the other hand, patients with clinical T4 tumors can be recommended to undergo SL [19]. Although there was no significant difference in P/CY-positive rate between T4 tumors and T3 ≥ tumors in our study, patients with T4 tumors tended to show higher P/CY-positive rates. The clinicopathological features of tumors are essential to deciding the indications for SL. However, the indication using preoperative laboratory parameters along with clinicopathological features has not been previously proposed. Here, we found that a higher NLR was significantly associated with a P/CY-positive result during SL. Combining the NLR and macroscopic tumor type could be more helpful in adjusting the selection of candidates for SL.

Recently, the NLR has been reported as a significant independent predictor of the presence of peritoneal metastasis in patients with advanced GC [20], and such finding is consistent with our results. The predictive value of the NLR for peritoneal metastasis during SL has been investigated in early gastric cancer or lower esophageal cancer, but not in advanced GC [21]. In our study, we demonstrated that a high NLR was a significant independent predictor of a P/CY-positive result during SL in patients with advanced GC. Most importantly, we found that preoperative NLR was associated with peritoneal disease spread regardless of tumor type. Although the macroscopic tumor features or severity of tumor invasion in preoperative examinations are very important for deciding the indication for SL, the NLR could be a supplemental criteria to decide whether SL is performed. In contrast, among patients with a low NLR without type 4 or diffuse type 3 tumors, none exhibited a P/CY-positive result. This result may suggest that patients with a low NLR without type 4 or diffuse type 3 tumors do not need to undergo SL for the evaluation of peritoneal metastasis and should be immediately planned for curative operation or neoadjuvant chemotherapy. Combining macroscopic tumor features and the NLR could be a promising strategy in further reducing the number of candidates for SL. Furthermore, we demonstrated the optimal cutoff value of the NLR to be 3.7, which is higher than the value in previous reports analyzing the predictive values of the NLR for the prognosis of GC [22, 23]. It might be reasonable that the cutoff value was set higher to predict peritoneal disease spread because our cohort consisted of the patients with more advanced GC who were indicated for SL. In the future, the optimal cutoff value for predicting peritoneal metastasis should be validated in a prospective study.

The NLR is a simple index of the systemic inflammatory response [24, 25]. Previously, several studies have reported that laboratory parameters reflecting inflammation, such as the NLR and serum CRP level, may be related to the prognosis of cancer, for example, GC, colorectal cancer, and lung cancer [12, 24, 26, 27]. Although it is unclear why the NLR could be elevated in cancer with a poor prognosis, these results suggest the existence of a close relationship between inflammation and cancer progression. Among inflammatory parameters, elevated serum levels of interleukin 6 (IL-6) but not CRP were reported to be associated with the presence of peritoneal metastasis in GC patients [28]. It was also reported that the interaction of neutrophils with tumor cells promoted GC cell migration and invasion through a pathway involving IL-6 [29]. Therefore, an increased neutrophil count may be attributed to an upregulation of IL-6 due to peritoneal disease progression. On the other hand, the immune response of the hosts to tumors depends on the lymphocytes [30], and an increased number of neutrophils suppresses the cytolytic activity of the lymphocytes [31, 32]. Considering these results, an increased neutrophil count and a decreased immune response to tumors may be related to the mechanism of peritoneal metastasis progression, and an increased NLR may reflect these conditions.

This study has certain limitations. It was a retrospective study performed at a single institution; additionally, the sample size was very small. Although we indicated that the NLR could have a strong potential as a predictor for the presence of peritoneal metastasis during SL, the current study lacks statistical strength due to the small sample size. In order to overcome the shortcomings of this study and confirm the predictive value of the NLR, we should perform a prospective cohort study in multiple institutions

## Conclusions

We found that the high preoperative NLR was associated with the presence of peritoneal metastasis during SL in patients with advanced GC. When adjusting the indication for SL, the NLR could be useful as an adjunct to the decision of criteria for SL by combining it with macroscopic tumor features.

## Data Availability

All data are available without restriction. Researchers can obtain data by contacting the corresponding author.

## Abbreviations

CA125: Carbohydrate antigen
CA19-9: Carbohydrate antigen 19-9
CEA: Carcinoembryonic antigen
CRP: C-reactive protein
GC: Gastric cancer
IL-6: Interleukin 6
NLR: Neutrophil/lymphocyte ratio
ROC: Receiver operating characteristic
SL: Staging laparoscopy
TP: Total protein
WBC: White blood cell
